# Exploring the Development of a Clean-Label Vegan Burger Enriched with Fermented Microalgae

**DOI:** 10.3390/foods14162884

**Published:** 2025-08-20

**Authors:** Joseane C. Bassani, Valter F. R. Martins, Joana Barbosa, Marta Coelho, Clara Sousa, Juliana Steffens, Geciane T. Backes, Hugo Pereira, Manuela E. Pintado, Paula C. Teixeira, Alcina M. M. B. Morais, Rui M. S. C. Morais

**Affiliations:** 1 CBQF—Centro de Biotecnologia e Química Fina—Laboratório Associado, Escola Superior de Biotecnologia, Universidade Católica Portuguesa, Rua Diogo Botelho, 1327, 4169-005 Porto, Portugal; joseanebassani@gmail.com (J.C.B.); vfmartins@ucp.pt (V.F.R.M.); jbarbosa@ucp.pt (J.B.); mcoelho@ucp.pt (M.C.); cssousa@ucp.pt (C.S.); mpintado@ucp.pt (M.E.P.); pcteixeira@ucp.pt (P.C.T.); 2 Departamento de Ciências Agrárias, Universidade Regional Integrada do Alto Uruguai e das Missões Uri Erechim, Erechim 99709-910, RS, Brazil; julisteffens@uricer.edu.br (J.S.); gtoniazzo@uricer.edu.br (G.T.B.); 3 GreenCoLab, University of Algarve, 8005-139 Faro, Portugal; hugopereira@greencolab.com

**Keywords:** microalgae, *Haematococcus pluvialis*, *Porphyridium cruentum*, color, volatile organic compounds, antioxidant activity, fermentation, *Lactiplantibacillus plantarum*, vegan burger, plant-based meat analogue (PBMA)

## Abstract

*Haematococcus pluvialis* and *Porphyridium cruentum* are red microalgae with high biotechnological potential due to their rich composition of bioactive compounds. However, their intense flavor limits their application in food products. This study evaluated the impact of fermentation with *Lactiplantibacillus plantarum* (30 °C for 48 h; LAB-to-biomass ratio of 0.1:1; 10^6^ CFU/mL) on the physicochemical and functional properties of *H. pluvialis* and *P. cruentum* biomasses. Particular attention was given to antioxidant activity (ABTS and ORAC assays), color, amino acid profiles, and volatile organic compound (VOC) profiles, all of which may influence sensory characteristics. Results demonstrated that non-fermented *H. pluvialis* exhibited significantly higher antioxidant activity (AA) than *P. cruentum*. After fermentation, *H. pluvialis* showed an ABTS value of 3.22 ± 0.35 and an ORAC value of 54.32 ± 1.79 µmol TE/100 mg DW, while *P. cruentum* showed an ABTS of 0.26 ± 0.00 and an ORAC of 3.11 ± 0.13 µmol TE/100 mg DW. Total phenolic content (TPC) of fermented *H. pluvialis* and *P. cruentum* was 1.08 ± 0.23 and 0.18 ± 0.026 mg GAE/100 mg DW, respectively. Both AA and TPC increased after fermentation. Fermentation also significantly affected biomass color. FTIR analysis showed intensification of protein and carbohydrate vibrational bands post-fermentation. GC-MS analysis of VOCs showed that *P. cruentum* contained 42 VOCs before fermentation, including *trans-*β-ionone, 4-ethyl-6-hepten-3-one, hexanal, and heptadienal, which are responsible for fishy and algal odors. Fermentation with *Lb. plantarum* significantly reduced these compounds, lowering *trans-*β-ionone to 0.1453 mg/L and eliminating 4-ethyl-6-hepten-3-one entirely. *H. pluvialis* contained 22 VOCs pre-fermentation; fermentation eliminated hexanal and reduced heptadienal to 0.1747 ± 0.0323 mg/L. These changes contributed to improved sensory profiles. Fermentation also induced significant changes in the amino acid profiles of both microalgae. The fermented biomasses were incorporated into vegan burgers made from chickpea, lentil, and quinoa. Color evaluation showed more stable and visually appealing tones, while texture remained within desirable consumer parameters. These findings suggest that *Lb. plantarum* fermentation is an effective strategy for improving the sensory and functional characteristics of microalgal biomass, promoting their use as sustainable, value-added ingredients in innovative plant-based foods.

## 1. Introduction

Microalgae have gained prominence as functional and sustainable food ingredients, representing a promising source of nutrients and bioactive compounds [1]. Among the many species, *Haematococcus pluvialis* and *Porphyridium cruentum* have emerged as particularly noteworthy due to their rich nutritional profiles, including proteins, polyunsaturated fatty acids, vitamins, minerals, and pigments, specifically astaxanthin in *H. pluvialis* and phycoerythrin in *P. cruentum* [2,3]. These compounds provide strong antioxidant potential and various health benefits, making these microalgae attractive to the pharmaceutical, nutraceutical, cosmetic, and food and beverage industries [4]. In addition to their nutritional benefits, microalgae offer advantages for sustainable large-scale production, including high biomass yields per unit area and the ability to grow without arable land [5,6]. They can be cultivated in closed systems using fewer natural resources and occupying less space than conventional crops. They thrive in controlled environments, such as photobioreactors, using only water and sunlight as the main inputs [5].

*Haematococcus pluvialis*, the primary natural source of astaxanthin, has achieved formal approval under the European novel food regulation. Specifically, astaxanthin-rich oleoresin derived from *H. pluvialis* was authorized as a novel food ingredient in food supplements within the EU Union list, with a permitted intake of 40–80 mg oleoresin (≤8 mg astaxanthin) per day for individuals aged 14 years and older [7,8,9]. In November 2023, EFSA reaffirmed the safety of this oleoresin, concluding that the acceptable daily intake (ADI) of 0.2 mg/kg body weight (bw) established for both synthetic and microalgae-derived astaxanthin also applies to this formulation [10,11]. In contrast, *Porphyridium cruentum* (synonym *P. purpureum*) has not yet been approved as a novel food by the EU and is not included on the Union list. It remains classified as a novel ingredient requiring submission of a full safety dossier. No EFSA opinions or Union list entries currently exist for *P. cruentum* or other *Porphyridium* species, despite growing academic interest.

In the United States, astaxanthin from *H. pluvialis* is permitted for limited uses; the U.S. FDA allows *Haematococcus* algae meal as a colorant in animal feed (e.g., salmon feed), but has not granted GRAS (Generally Recognized As Safe) status for direct human consumption of *H. pluvialis* biomass or oleoresin. Likewise, no GRAS affirmation or notification is publicly available for *Porphyridium* species in a food context. However, *H. pluvialis* and *Porphyridium* spp., including *P. cruentum*, have been mentioned in the literature as part of the group deemed “GRAS” by the FDA for specific uses such as pigment extraction, namely astaxanthin and phycoerythrin, respectively [12,13]. Peer-reviewed safety data provide additional context. Acute and subchronic toxicity studies in rats show that *H. pluvialis* biomass and astaxanthin-rich extracts are well tolerated, with LD_50_ values exceeding 12 g/kg bw and no significant adverse effects on hematology or histopathology at dietary levels up to 20% for 90 days (e.g., LD_50_ > 5000 mg/kg; mild renal pigment changes deemed non-toxicologically relevant) [14,15]. For *P. cruentum*, an OECD-compliant genotoxicity and 90-day oral toxicity study in rats reported no mutagenic or chromosomal damage, with a no-observed-adverse-effect level (NOAEL) established at 3000 mg/kg bw/day [16]. In aquatic models, exopolysaccharide (EPS) extracts caused no mortality or developmental abnormalities in zebrafish embryo assays at exposures up to 15% over 96 h. In vitro cytotoxicity assays showed low cytotoxicity from most EPS fractions, except for specific acidified preparations at very high concentrations [17,18].

Despite their high potential and numerous benefits, the acceptance of microalgae as food ingredients is still limited due to their undesirable sensory characteristics, such as strong flavors and odors often described as “fishy” or “seaweedy” [19]. These negative characteristics are largely attributed to volatile organic compounds (VOCs), including aldehydes and ketones, produced through different metabolic pathways during the microalgae’s life cycle [20]. To facilitate broader adoption and consumer acceptance, microalgae must undergo improvements that enhance their sensory appeal and position them as viable crop alternatives for food applications [6]. Therefore, strategies that effectively modulate and improve the sensory profile of microalgae are essential. Microbial fermentation has emerged as a promising approach to transform and enhance various food raw materials without generating toxic residues [21,22].

Lactic acid bacteria (LAB), such as *Lactiplantibacillus plantarum* (former *Lactobacillus plantarum*), are being widely used in food fermentation processes due to their ability to metabolize carbohydrates, proteins, and lipids, producing organic acids, enzymes, and other metabolites that improve flavor, aroma, and functional properties [23]. LAB fermentation is also effective at reducing undesirable compounds and generating new ones that contribute to a more pleasant sensory profile [24].

This study aimed to investigate the impact of fermentation with *Lactiplantibacillus plantarum* on the sensory and functional properties of *H. pluvialis* and *P. cruentum* biomasses. Specifically, we quantified antioxidant activity and total phenolic content, evaluated color changes, and conducted VOC profiling to assess the effects of fermentation on sensory-relevant volatile composition. Additionally, this preliminary study explored the formulation and characterization of a clean-label vegan burger incorporated with fermented microalgae to demonstrate their feasibility as sustainable, value-added ingredients and innovative plant-based foods.

## 2. Materials and Methods

All chemical compounds were p.a. grade.

### 2.1. Microalgae Biomass and Cultivation Conditions

*Haematococcus pluvialis* (Yunnan Alcom Biotech Co, Ltd., Chuxiong, China) and *Porphyridium cruentum* biomasses were provided by GreenCoLab (Faro, Portugal). The *P. cruentum* strain was grown under controlled light, temperature, and nutrient conditions, and then harvested by centrifugation (5000 rpm, 15 min) (sample A). Sample B of *P. cruentum* was kindly donated frozen by A4F—Algae for Future, Portugal. All biomasses were stored at −20 °C until use, when they were defrosted at 4 °C overnight. A part of the biomass was freeze-dried.

### 2.2. Fermentation Procedure of the Biomass

The fermentation protocol was developed initially based on a literature review [25], but mostly through preliminary experimental trials. To improve product yield and process efficiency, we conducted a series of bench-scale trials testing biomass concentrations of 10%, 20%, 30%, and 40% (*w*/*v*). The 40% *w*/*v* microalgae suspension was selected based on its ability to maximize substrate availability while maintaining suitable rheological properties (no excessive viscosity), effective mixing, and stable fermentation without compromising microbial viability or conversion efficiency.

Commercial strains of *Lactiplantibacillus plantarum* were individually suspended in Ringer’s solution (Biokar diagnostics, Paris, France). The powdered biomasses of *H. pluvialis* and *P. cruentum* were resuspended in sterile distilled water to obtain a 40% (*m*/*v*) suspension. Individual fermentations of the biomasses were carried out in sterile bags containing the microalgae, inoculated with the microorganism at a ratio of 0.1 LAB:1 biomass (culture volume: biomass fresh weight), to a final concentration of approximately 10^6^ CFU/mL. After homogenization, the samples were incubated at 30 °C for 48 h without agitation. The pH varied between 5.9 ± 0.45 and 6.8 ± 0.3 in the *H. pluvialis* fermentation medium, and 4.8 ± 0.3 and 6.2 ± 0.4 for *P. cruentum.* Non-fermented microalgae samples served as controls. After fermentation, the biomass was freeze-dried for subsequent use.

The samples were identified as

H: *H. pluvialis* biomass.

P: *P. cruentum* biomass.

FH: *H. pluvialis* fermented biomass with *Lb. plantarum.*

FP: *P. cruentum* fermented with *Lb. plantarum.*

### 2.3. Physicochemical Analyses

#### 2.3.1. Determination of Amino Acid Profiles

- Sample Preparation

A 10 mg sample (freeze-dried) was transferred into a vial for solid-phase microextraction (SPME) and mixed with 3 mL of 6 M HCl (Sigma-Aldrich, Saint Louis, MO, USA). The mixture was vortexed, purged with nitrogen gas for 4 min, sealed with tape, and incubated in an oven at 115 °C overnight. After cooling to room temperature, 4 mL of Milli-Q water was added, and the pH was adjusted to 3.5 using 10 M NaOH. The final volume was brought to 10 mL with Milli-Q water, and the solution was filtered through a 0.45 μm membrane filter.

- HPLC Analysis

The qualitative and quantitative profiling of amino acids was performed according to the method described by Long [26]. Briefly, 100 µL of a filtered sample solution (10 mg/mL freeze-dried hydrolysate) was mixed with derivatization reagents, and a 10 µL aliquot of the reaction mixture was subsequently injected into the HPLC system. Sample preparation is detailed above. Chromatographic analysis was carried out using an Agilent 1200 Series HPLC system (Agilent Technologies, Santa Clara, CA, USA), equipped with an LC-20AB binary pump, SIL-20A autosampler, CTO-20A column oven maintained at 25 °C, and a G1321A fluorescence detector (FLD) operating at an excitation wavelength of 356 nm and an emission wavelength of 445 nm. Data acquisition and processing were managed using the LC Ver1.23 workstation software (Agilent Technologies). Separation of amino acids was achieved using an Agilent Poroshell HPH-C18 column (2.1 × 200 mm, 5 µm particle size), employing a method adapted from Long [26]. A Hypersil AA-ODS column (Agilent Technologies) served as the stationary phase. The mobile phase A consisted of 10 mM Na_2_HPO_4_, 10 mM Na_2_B_4_O_7_, and 5 mM NaN_3_ (Sigma-Aldrich, Saint Louis, MO, USA), adjusted to pH 8.4 with concentrated HCl and filtered through 0.45 µm regenerated cellulose membranes (p/n 3150-0576). Mobile phase B comprised a mixture of acetonitrile, methanol, and water (45:45:10, *v*/*v*/*v*; Fisher Chemical, Pittsburgh, PA, USA). A gradient elution was applied as follows: starting from 90% A to 50% A over 43 min, increasing to 100% B between 47 and 49 min, and re-equilibrating to 90% A by 50 min. The flow rate was maintained at 0.9 mL/min throughout the run. Calibration standards for the following amino acids were prepared at a concentration of 100 mg/L: alanine, asparagine, aspartic acid, cysteine, glutamic acid, glutamine, glycine, isoleucine, leucine, methionine, valine, proline, serine, threonine, phenylalanine, tryptophan, and tyrosine. All standards were obtained from Sigma Chemical Co. (Saint Louis, MO, USA).

#### 2.3.2. Analysis of Volatile Organic Compounds (VOCs)

Volatile organic compounds (VOCs) were analyzed using gas chromatography coupled with mass spectrometry (GC-MS) on a 456-GC EVOQ TQ system (Bruker, Billerica, MA, USA), employing solid-phase microextraction (SPME). Approximately 0.5 g of *Haematococcus pluvialis* and 1.0 g of *Porphyridium cruentum*—both fermented and non-fermented fresh samples—were combined with 4 mL of distilled water, 1 g of sodium chloride (NaCl), and 10 µL of a 5.0 mg/L solution of 3-octanol (Sigma-Aldrich, Saint Louis, MO, USA), used as an internal standard.

Samples were incubated at 60 °C for 20 min under agitation. Following incubation, the SPME fiber (Supelco, Bellefonte, PA, USA) was exposed to the sample headspace at the same temperature for 30 min, then inserted into the GC injector for thermal desorption at 250 °C for 5 min.

Compound identification was conducted using the NIST mass spectral database. The concentrations of VOCs were calculated based on the peak area relative to the internal standard and expressed in mg/L.

#### 2.3.3. Determination of the Total Phenolic Content (TPC)

Extracts were prepared from each lyophilized biomass sample (fermented and non-fermented) by mixing with methanol in a ratio of 0.6:10.0 (*w*/*v*). The mixture was subjected to continuous agitation using a ROLLER 10 digital shaker (IKA, Staufen, Germany) at 60 rpm for 16 h.

The total phenolic content (TPC) of the extracts was determined using the Folin–Ciocalteu method, following the procedure described by Martins et al. [27]. Briefly, 100 μL of Folin–Ciocalteu reagent (20% *v*/*v*) was added to 30 μL of the methanolic extract. Subsequently, 100 μL of sodium carbonate solution (7.4% *w*/*v*) was added, and the mixture was incubated in the dark at room temperature (approximately 25 °C) for 30 min. Absorbance was measured at 765 nm using a Synergy H1 microplate reader (BioTek, Winooski, VT, USA) in 96-well microplates (Sarstedt, Nümbrecht, Germany). Gallic acid was used as the standard for calibration, and results were expressed as milligrams of gallic acid equivalents per 100 mg of dried extract (mg GAE/100 mg DW). All measurements were performed in triplicate in three independent experiments.

#### 2.3.4. Antioxidant Activity Determination

The antioxidant activity of the extract solutions prepared above (0.6:10.0 *w*/*v*) was determined using three different methods

- ABTS Radical-Scavenging Assay

The ABTS [2,2′-azino-bis(3-ethylbenzothiazoline-6-sulfonic acid)] radical-scavenging activity was assessed following the protocol described by Martins et al. [27]. Briefly, the ABTS•^+^ radical cation was generated by chemical oxidation of ABTS salt (0.0384 g in 10 mL of ultrapure water) with potassium persulfate (0.0066 g in 10 mL of ultrapure water) and incubated in the dark at room temperature (approximately 25 °C) for 16 h prior to use. The resulting solution was then diluted with ultrapure water to an initial absorbance of 0.700 ± 0.020 at 734 nm using a microplate spectrophotometer (Synergy H1, Biotek, Winooski, VT, USA). For the assay, 20 μL of each methanolic extract was added to 180 μL of the diluted ABTS•^+^ solution in 96-well microplates (Sarstedt, Nümbrecht, Germany) and incubated in the dark at room temperature for exactly 5 min. Methanol was used as the blank. The absorbance was measured at 734 nm.

The percentage of inhibition (I) was calculated using Equation (1):

I (%) = [(Abs_A0 − Abs_sample)/Abs_A0] × 100(1)

where Abs_A0 is the absorbance of the control and Abs_sample is the absorbance in the presence of the extract.

A Trolox calibration curve was used to express the results as micromoles of Trolox equivalents per 100 mg of dried extract (μmol TE/100 mg DW). All assays were performed in triplicate in three independent experiments.

- DPPH Radical-Scavenging Assay

The antioxidant capacity of the extracts was also assessed using the DPPH [2,2-diphenyl-1-picrylhydrazyl] radical-scavenging method, following the procedure described by Martins et al. [27]. Briefly, a stock solution of DPPH in methanol (600 μM) was prepared and stored at −20 °C in the dark. A working solution of 90 μM was freshly prepared by diluting 15 mL of the stock solution with 85 mL of methanol to yield an initial absorbance of 0.600 ± 0.100 at 515 nm. In a 96-well microplate (Sarstedt, Nümbrecht, Germany), 25 μL of the methanolic extract was mixed with 175 μL of the DPPH working solution and incubated in the dark at room temperature (approximately 25 °C) for 30 min. Absorbance was then measured at 515 nm using a microplate reader (Synergy H1, BioTek, Winooski, VT, USA), with methanol serving as the blank.

The percentage of radical inhibition (I) was calculated according to Equation (1). A Trolox calibration curve was used to quantify the antioxidant capacity, and results were expressed as micromoles of Trolox equivalents per 100 mg of dried extract (μmol TE/100 mg DW). All assays were performed in triplicate across three independent experiments.

- Oxygen Radical Absorbance Capacity (ORAC) Assay

The antioxidant capacity of the extracts was determined using the oxygen radical absorbance capacity with fluorescein (ORAC-FL) assay, performed in black 96-well microplates (Thermo Scientific, Roskilde, Denmark), following the protocol described by Martins et al. [27]. Briefly, 20 µL of each extract was mixed with 120 µL of fluorescein (final concentration of 70 nM) and 60 µL of 2,2′-azobis(2-amidinopropane) dihydrochloride (AAPH). Control wells contained 80 µL of phosphate buffer (75 mM, pH 7.4) and 120 µL of fluorescein solution, while blank wells contained fluorescein and AAPH, with phosphate buffer in place of the extract. A Trolox standard curve was prepared using eight concentrations (1–8 µM final concentration per well). The microplate was pre-incubated at 37 °C for 10 min. Subsequently, 60 µL of AAPH (final concentration 12 mM) was added rapidly to initiate the reaction. Fluorescence was measured every minute for 90 min using a multi-mode microplate reader (Synergy H1, BioTek Instruments, Winooski, VT, USA), with excitation at 485 nm and emission at 528 nm. Data acquisition was performed using Gen5 software (v3.04).

All reagents were freshly prepared on the day of analysis. Fluorescein was diluted from a 1.17 mM stock solution in 75 mM phosphate buffer (pH 7.4). Fluorescence decay curves were normalized by multiplying the raw fluorescence values by the blank fluorescence at time zero and dividing by the control fluorescence at time zero. The area under the curve (AUC) was calculated for each well, and net AUCs were obtained by subtracting the blank AUC. Results were expressed as micromoles of Trolox equivalents per 100 mg of dry extract (µmol TE/100 mg DW). All measurements were performed in triplicate across three independent experiments.

#### 2.3.5. Mid-Infrared (MIR) Spectra of Fermented and Non-Fermented Microalgae

Mid-infrared (MIR) spectra of freeze-dried fermented and non-fermented microalgae were obtained using a PerkinElmer Spectrum BX FTIR spectrophotometer (PerkinElmer, Waltham, MA, USA), equipped with a deuterated triglycine sulfate (DTGS) detector and Fourier transform capabilities. Spectra were acquired in diffuse reflectance mode using a Gladi Attenuated Total Reflectance (ATR) accessory (PIKE Technologies), in the wavenumber range of 4000–600 cm^−1^ with a spectral resolution of 4 cm^−1^. Each spectrum was the result of 32 co-added scans. Freeze-dried microalgae samples were directly placed onto the ATR crystal, and consistent pressure was applied to ensure uniform contact. The crystal was thoroughly cleaned between each sample, and a new background spectrum was recorded before each acquisition. Three replicate spectra were obtained for each sample.

Spectral data were analyzed using principal component analysis (PCA), as originally described by Jolliffe [28], to explore the biochemical variation between samples. Prior to modelling, the spectral data were preprocessed using standard normal variate (SNV) normalization [29], followed by a Savitzky–Golay second derivative filter (15 smoothing points, second-order polynomial) [30]. The processed data were mean-centered before PCA. All preprocessing and multivariate modelling procedures were conducted using MATLAB R2023a (MathWorks, Natick, MA, USA) in combination with PLS Toolbox version 9.2.1 (Eigenvector Research, Manson, WA, USA).

#### 2.3.6. Color Evaluation

The color parameters of the fermented and non-fermented microalgal biomasses (fresh) were measured using a colorimeter (Chroma Meter CR-400, Konica Minolta, Tokyo, Japan) based on the CIE Lab system [31]. In this color space, L* indicates lightness (ranging from 0 = black to 100 = white), a* represents the green (−) to red (+) axis, and b* represents the blue (−) to yellow (+) axis. Hue angle (°) and chroma (C*) were calculated using the following formulas:

Hue = arctangent (b*/a*)(2)

(3) $$C*=\sqrt{a{*}^{2}+b{*}^{2}}$$

Ten independent measurements (*n* = 10) were conducted for each biomass sample to ensure statistical robustness.

### 2.4. Application in Vegan Burgers

The formulation of a nutritionally enhanced vegan burger involved systematic trials to identify the optimal combination of plant-based ingredients and microalgal biomass. Chickpeas, lentils, and quinoa were selected as the core base components due to their complementary nutritional and functional attributes: quinoa contributes protein and texture, chickpeas provide creaminess and serve as a binding agent, and lentils supply fiber and structural integrity, thereby improving consistency and sensory quality [32]. To enhance the nutritional profile and incorporate bioactive compounds, both fermented and non-fermented microalgal biomasses, *Haematococcus pluvialis* (4% *w*/*w*) and *Porphyridium cruentum* (6% *w*/*w*), were integrated into the final formulation. These concentrations were optimized to balance nutritional enrichment with desirable visual, textural, and handling properties. Preliminary trials with varying biomass levels (1%, 3.5%, 6%, and 12% *w*/*w*) demonstrated that formulation choice critically influenced post-baking texture, with chickpea-based burgers becoming excessively pasty and lentil-based burgers overly dry, highlighting the importance of precise ingredient ratios for product integrity. Therefore, fermented and non-fermented microalgal biomasses were incorporated into the base mixture according to different formulations (*w*/*w*):

C (control): 50% chickpeas, 20% lentils, 20% quinoa; 6% corn oil; 2% oat flour; 2% condiments;

HB: 4% non-fermented *H. pluvialis*; 96% C;

PB: 6% non-fermented *P. cruentum*, 94% C;

HPB: 3% *H. pluvialis* and 3% *P. cruentum,* both non-fermented, 94% C;

FHB: 4% *H. pluvialis* fermented with *Lactobacillus plantarum*, 96% C;

FPB: 6% *P. cruentum* fermented with *Lactobacillus plantarum*, 94% C;

FHPB: 3% *H. pluvialis* and 3% *P. cruentum* both fermented with *Lactobacillus plantarum*, 94% C.

Each burger weighed 125 g.

The nutritional composition, as well as the color, texture, and pH of the vegan burgers, were subsequently analyzed.

#### 2.4.1. Nutritional Characterization of the Vegan Burgers

The dry matter, moisture, ash, fat, protein, fiber, and carbohydrate contents and the energetic value of the solid residue after the PS extraction were determined.

- Moisture Content

About 1 g of sample was placed in a Petri dish inside an oven at 105 °C for 24 h and subsequently weighed [33]. Three replicates were performed. The moisture content was calculated through the following equation:

Moisture content (%) = (weight at time 0 − weight of dried sample)/weight at time 0 × 100(4)

- Ash

About 2.5 g of the sample was incinerated in a muffle furnace at 550 °C ± 15 °C until constant weight [34].

- Total fat

About 10 g of the sample was boiled in a beaker (in reflux conditions, with dilute hydrochloric acid 4 M for 30 min) and filtered. The fat was extracted for 4 h with petroleum ether in a Soxhlet extractor into a previously weighed round-bottom flask. The solvent was evaporated in a rotary evaporator (Buchi R-210, Buchi Labortechnik AG, Flawil, Switzerland), and the residue was dried to a constant weight at 102 ± 2 °C [34].

- Saturated fat

Saturated fat content was determined by gas chromatography with a flame ionization detector (GC-FID) following lipid extraction and methylation, as described by AOAC Official Method 996.06 [35].

- Protein

The protein content was determined using ISO 1871:2009 [36]. Briefly, about 0.5 g of sample was digested in a mineralization block (Kjeltec Foss, Burladingen, Germany), with concentrated sulfuric acid (96% *w*/*w*) in the presence of a catalyst. From the quantity of ammonia produced, the nitrogen content (N) was calculated. The protein was calculated using the following formula:

% protein = % N × 6.25(5)

At the same time, a blank test determination was performed.

- Total dietary fiber

Briefly, the methods used were AOAC 991.43 [37] and AOAC 985.29 [38], and the assay was performed in duplicate. About 1 g of the sample was submitted to enzymatic digestion using three enzymes: α-amylase (30 min at 90 °C), followed by protease (30 min at 60 °C), followed by amyloglucosidase (30 min at 60 °C). To obtain the insoluble and soluble fibers in the first filtration (using celite), the residue was cleaned with 10 mL of water at 70 °C, and then with 10 mL of 95% ethanol and 10 mL of acetone. To determine the soluble fiber, four volumes of ethanol pre-heated at 60 °C were added, and the residue was washed with 15 mL of 75% ethanol, 95% ethanol, and acetone. This residue was dried, and the protein and ash contents were determined, the remainder being soluble fiber, subtracting the weight of celite. The total fiber was calculated by adding the insoluble and soluble fiber contents.

- Total carbohydrates

The total carbohydrates were determined by differences according to the European Commission [39] and using the following formula:

Carbohydrates = 100 − (moisture + ash + fat + protein)(6)

- Sodium concentration

Sodium concentration was measured using flame atomic absorption spectroscopy (FAAS) following AOAC Official Method 985.35 [40].

- NaCl

Sodium chloride (NaCl) content was determined indirectly by measuring sodium concentration via flame atomic absorption spectroscopy (FAAS) and calculating NaCl content using a conversion factor, as described in AOAC Official Method 937.09 [38].

- pH

The pH was measured potentiometrically at 20 °C using a calibrated pH meter, following the ISO 2917:1999 standard [41].

- Determination of the Energetic Value

For the determination of the energetic value, the following formula (Regulation (EU) No. 1169/2011) [39] was used:

Energetic value (kcal/100 g sample) = 4 × (mass of protein + mass of carbohydrates) + 9 × mass of fat(7)

#### 2.4.2. Burger Color Evaluation

Color measurements of the burger patties were carried out as described previously in Section 2.3.6, using the CIE Lab system.

#### 2.4.3. Burger Texture Evaluation

The choice of test parameters, setup, and conditions for the instrumental texture evaluation was guided by the need to accurately characterize the mechanical and structural properties of different formulations, particularly in the context of alternative meat products. The selected parameters, hardness, elasticity, and chewiness, and the experimental setup were well-suited to differentiate between the textural profiles of complex food matrices’ attributes. These parameters were chosen because they are critical to the sensory perception and consumer acceptance of meat alternatives. Hardness reflects how firm or soft the product feels during the first bite. Elasticity indicates how well the product returns to its shape, mimicking the fibrous and resilient texture of meat. Chewiness combines hardness and elasticity to describe the effort that is required to chew the product, influencing the overall mouthfeel. Together, these parameters enable differentiation between complex food matrices and provide objective data to guide the optimization of meat alternative formulations [42].

The texture was assessed using a texture analyzer (TA.XTPlus, Stable Micro Systems, Godalming, UK) employing the Texture Profile Analysis (TPA) method [42]. Measurements were conducted at a crosshead speed of 0.3 mm/s, with 50% compression of the burger height and a 1.0 s interval between two compression cycles. A cylindrical probe (10 mm diameter) was used. Texture analyses were performed in nine replicates (*n* = 9).

The following texture parameters were then evaluated:

- Hardness: maximum force (Newton, N) required for sample compression [43].
- Elasticity (springiness): ability of the sample to recover its original shape after compression (dimensionless) [44].
- Chewiness: amount of energy required to chew the sample until it is ready for swallowing, calculated in N [45].

### 2.5. Statistical Analysis

All experiments were conducted in triplicate, and the results were expressed as the mean ± standard deviation (SD). Data were subjected to one-way analysis of variance (ANOVA), followed by Tukey’s post hoc test to determine significant differences between means (*p* < 0.05). Statistical analyses were performed using Statistica software version 10.0 (StatSoft, Inc., Tulsa, OK, USA). To summarize and interpret the data, a principal component analysis (PCA) was conducted.

## 3. Results

### 3.1. Amino Acid Profiles of the Fermented and Non-Fermented Biomasses

Table 1 presents the mean concentrations (mg/100 mg) and standard deviations of amino acids in the four algal biomass samples FH (*Haematococcus* fermented), FP (*Porphyridium* fermented), P (*Porphyridium* non-fermented), and H (*Haematococcus* non-fermented). The fermentation process had a marked effect on both total amino acid concentration and specific amino acid profiles.

The total amino acid (AA) content varied significantly across samples (*p* < 0.01), with H presenting the highest value (34.8 mg/100 mg), which was substantially greater than that of FH (25.7 mg/100 mg). This suggests that the fermentation of *Haematococcus* led to a substantial reduction in total amino acids, likely due to microbial catabolism of proteins or amino acids during the fermentation. Lactic acid bacteria (LAB), including *Lactiplantibacillus plantarum*, are capable of utilizing free amino acids not only as nitrogen sources but also, under certain conditions, as carbon and energy sources through deamination, decarboxylation, or transamination pathways [46,47]. Notably, *Lb. plantarum* can metabolize branched-chain, sulfur-containing, and aromatic amino acids via specific enzymatic pathways, contributing to energy production and formation of bioactive or aroma compounds [48]. This metabolic activity supports the hypothesis that the amino acids in *H. pluvialis* biomass were actively consumed during fermentation. This catabolism may result in the generation of several metabolites, such as organic acids (e.g., lactic acid and acetic acid), short-chain fatty acids, bioactive peptides, volatile compounds (e.g., aldehydes, alcohols, and esters), and γ-aminobutyric acid (GABA), via glutamate decarboxylation, which has known health benefits [25,49,50].

In contrast, FP (0.287 mg/100 mg) showed a slight but statistically significant increase compared to P (0.272 mg/100 mg), suggesting that *Porphyridium* may respond differently to fermentation. This difference could be attributed to its unique cell wall composition or species-specific interactions with microbial metabolism.

The ratio of essential amino acids (EAA) to total amino acids remained relatively stable among samples, ranging from 58.11% in FH to 60.13% in FP, with no statistically significant differences observed (*p* > 0.05).

Among individual amino acids, valine, isoleucine, and glutamic acid were the most abundant. Significant differences were also observed in individual amino acids, including isoleucine, lysine, and alanine, indicating fermentation-induced shifts in specific amino acid profiles.

### 3.2. Volatile Organic Compounds (VOCs) Profiles

The analysis of volatile organic compounds (VOCs) provided crucial insights into sensory changes induced by fermentation.

#### 3.2.1. *Haematococcus pluvialis*

A total of 22 VOCs were identified in the non-fermented *H. pluvialis* biomass, including compounds such as hexanal and heptadienal, which are commonly associated with fishy and seaweed-like odors. Fermentation led to a reduction in several VOCs linked to undesirable odors, notably hexanal, which decreased to 0.3115 mg/L, and heptadienal, which was reduced to 0.4189 mg/L. These aldehydes, products of lipid oxidation, are known contributors to rancid or “green” off-flavors. Their reduction indicates an improvement in the overall aromatic profile. Table 2 presents the results of the main compounds associated with off-odors. The observed decrease in off-flavor VOCs suggests an enhanced sensory profile, thereby improving the potential of *H. pluvialis* biomass for incorporation into food products.

#### 3.2.2. *Porphyridium cruentum*

A total of 42 VOCs were identified in the non-fermented *P. cruentum* biomass, including compounds such as *trans*-β-ionone, 4-ethyl-6-hepten-3-one, hexanal, and heptadienal, which are commonly associated with fishy and seaweed-like odors. Fermentation with *Lactiplantibacillus plantarum* resulted in a notable reduction in these odor-contributing compounds. Specifically, the concentration of *trans*-β-ionone decreased to 0.1453 mg/L, while 4-ethyl-6-hepten-3-one was completely eliminated. This reduction significantly contributes to the attenuation of the characteristic flavor and odor typically associated with microalgae.

Table 3 presents the concentrations of the main compounds related to undesirable odors.

### 3.3. Total Phenolic Content and Antioxidant Activity of H. pluvialis and P. cruentum Before and After Fermentation

The results indicate that non-fermented *H. pluvialis* exhibited significantly higher antioxidant activity (as measured by ABTS and ORAC assays) and total phenolic content (TPC) compared to non-fermented *P. cruentum* (Table 4). Fermentation with *Lactiplantibacillus plantarum* led to a significant increase in both antioxidant activity and TPC in both microalgal species. Specifically, for *H. pluvialis*, the ABTS value increased from 2.3967 ± 0.1299 to 3.2185 ± 0.3526 µmol Trolox equivalents (TE)/100 mg dry weight (DW), while the ORAC value rose from 43.3276 to 54.3239 µmol TE/100 mg DW. The TPC also increased, from 0.1475 to 1.0795 mg gallic acid equivalents (GAE)/100 mg DW. Similarly, although the absolute values were lower in *P. cruentum*, fermentation still resulted in measurable increases: ABTS increased from 0.2644 to 0.3251 µmol TE/100 mg DW, ORAC from 1.3806 to 3.1111 µmol TE/100 mg DW, and TPC from 0.0816 to 0.1802 mg GAE/100 mg DW. The DPPH assay could not be determined for *H. pluvialis* biomass due to color interference from the extracts.

### 3.4. FTIR Spectra of Lyophilized Microalgae

Fourier-transform infrared (FTIR) spectroscopy was employed to investigate macromolecular alterations in proteins, lipids, and carbohydrates within microalgal biomass, with a focus on changes induced by fermentation. This technique, widely recognized for monitoring biochemical composition in complex biological matrices, enables the detection of subtle yet biologically meaningful shifts in structural and storage biomolecules. For *Haematococcus pluvialis*, both vegetative and aplanospore stages were analyzed in their unfermented form to establish a compositional baseline, thereby facilitating the interpretation of fermentation-induced spectral modifications; fermentation was applied exclusively to the industrially relevant aplanospore stage, characterized by high astaxanthin content. For *Porphyridium cruentum*, FTIR spectra were acquired from two cultivation sources (samples A and B) to assess the influence of growth conditions on biochemical architecture; no significant macromolecular differences were detected, and sample A, cultivated by the study’s authors, was selected for subsequent fermentation experiments. Overall, the FTIR approach provided mechanistic insight into the biochemical transformations associated with microalgal fermentation, offering a framework for linking cultivation stage, biomass composition, and fermentation outcomes [51].

#### 3.4.1. *Haematococcus pluvialis*

**Figure 1:**
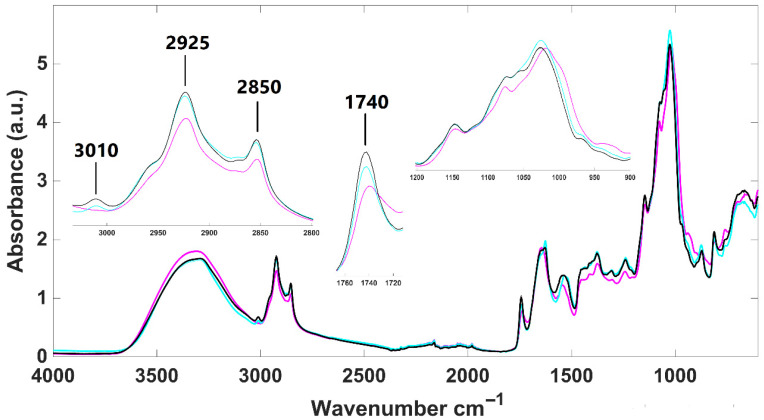
Infrared spectra of lyophilized *H. pluvialis* samples. Palmella (purplemagenta line); aplanospore (bluecyan line); fermented aplanospore (black line).

Infrared spectra of lyophilized *H. pluvialis* samples were obtained for the microalga in two different development stages, palmella and aplanospore, as well as for aplanospore after fermentation (Figure 1). Overall, the spectra exhibit typical absorption bands corresponding to main biological molecules: lipids (3050–2800 cm^−1^), proteins (1700–1500 cm^−1^), phospholipids/DNA/RNA (1500–1200 cm^−1^), and carbohydrates (1200–900 cm^−1^).

Aplanospore samples, whether fermented or not, appear to have a higher lipidic content than palmella ones, as indicated by more intense absorption bands at 2925 cm^−1^ and 2850 cm^−1^. These samples also show stronger signals for carotenoids, primarily astaxanthin, evidenced by the presence of a specific absorption band at 3010 cm^−1^, which is absent in palmella samples’ infrared spectra (zoom-in view in Figure 1). Additionally, all *H. pluvialis* infrared spectra present an absorption band around 1740 cm^−1^, corresponding to the ester carbonyl group in pure astaxanthin, as demonstrated by Liu and Huang [52]. This band exhibits greater intensity in aplanospore samples (fermented or not), suggesting higher astaxanthin content in this development stage. Distinct patterns were also observed in the carbohydrate region (1200–900 cm^−1^), reflecting structural differences in sugars between vegetative (palmella) and aplanospore phases. These findings are consistent with those of Guo et al. [53], who reported that cellulose present in flagellate (palmella) cells gradually transforms into mannose in the aplanospore stage.

**Figure 2:**
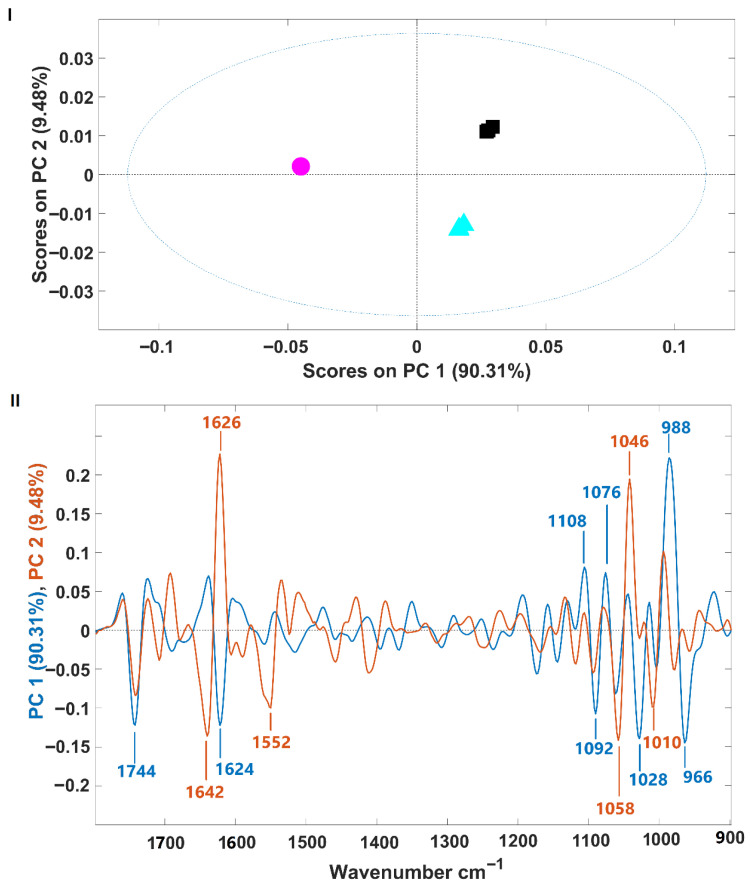
(**I**)—Scores plot from principal component analysis of infrared spectra of *H. pluvialis* samples in palmella and aplanospore stages (fermented or not). Palmella samples are shown as magenta circles; aplanospore samples are cyan triangles); fermented aplanospore samples are black squares; (**II**)—Loading plot of PC1 (blue, 90.31% variance) and PC2 (red, 9.48% variance), showing the wavenumber regions contributing most to sample separation.

Spectra were further analyzed using principal component analysis (PCA). The PCA score plot and corresponding loading plot are presented in Figure 2I and Figure 2II, respectively. The samples are clearly separated along the first two principal components (PCs), which together account for 99.79% of the spectral variance (PC1: 90.31%, PC2: 9.48%) (Figure 2I). *H. pluvialis* samples in the palmella stage (growth phase) are separated from those in the aplanospore stage (fermented or not) along the first principal component (PC1). Meanwhile, fermented and non-fermented aplanospore samples are distinguished along the second principal component (PC2). The intensity of the PCA loading (Figure 2II) provides insight into the spectral regions that contribute most to sample differentiation (i.e., regions with higher intensity have greater impact). For PC1, which separates palmella and aplanospore stages, the highest intensities are observed in the carbohydrate absorption region (from 1200 to 900 cm^−1^), suggesting significant differences in the quantity and structure of carbohydrates between these two development stages. This observation is consistent with previously discussed changes in sugar composition during the transition from flagellated (palmella) to aplanospore cells in *H. pluvialis*. For PC2, which differentiates between fermented and non-fermented samples, key spectral regions include bands at 1626 cm^−1^ and 1046 cm^−1^ (positively associated with fermented samples), and 1642 cm^−1^, 1552 cm^−1^, 1058 cm^−1^, and 1010 cm^−1^ (favorably associated with non-fermented samples). Some of these bands fall within the protein region (1700–1500 cm^−1^), while others lie within the carbohydrate region. Due to the biochemical complexity of microalgae biomass, precise band assignment remains challenging, as multiple compounds may contribute to vibrational signals in these wavenumber ranges. This includes VOCs, some of which are associated with the characteristic fishy odor of microalgae.

#### 3.4.2. *Porphyridium cruentum*

**Figure 3:**
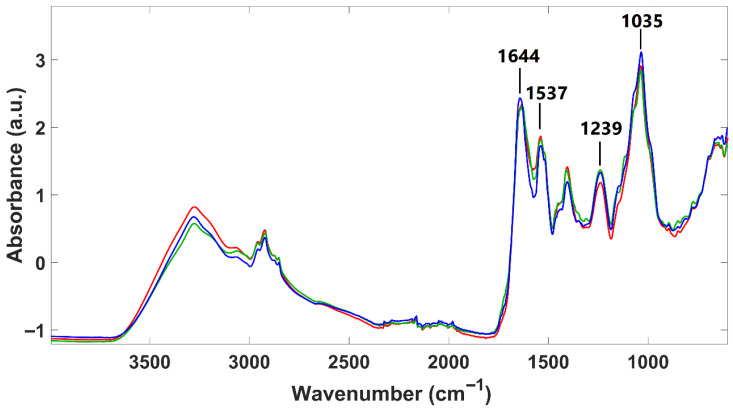
Infrared spectra of lyophilized *P. cruentum* samples. Sample A (blue line); sample B (red line); fermented A sample (green line).

Infrared spectra of lyophilized *P. cruentum* samples were obtained from two different culture sources (A and B) and, additionally, for sample A after fermentation (Figure 3). Overall, the spectra exhibit similar absorption bands from the main biological molecules, as reported for *H. pluvialis*. However, some notable differences between these two species were found. *H. pluvialis* appear to have a higher lipid and sugar content (indicated by more intense typical absorption bands) and a lower protein content (indicated by less intense absorption bands). Regarding the two microalgal culture origins (A and B fermented or not), only minor spectral differences are observed in their infrared profiles, suggesting that all *P. cruentum* samples analyzed here have quite a similar composition. Indeed, Fuentes-Grünewald et al. [54] identified, for *Porphyridium purpureum*, vibration bands located at 1035 cm^−1^, attributed to polysaccharides; at 1239 cm^−1^, attributed to nucleic acids and phospholipids; at 1537 cm^−1^ and 1644 cm^−1^, attributed to amide II and amide I, respectively. The wavenumbers identified by these authors precisely match those identified here for *P. cruentum*, suggesting a strong similarity in macromolecular composition among individuals from the *Porphyridium genus*.

**Figure 4:**
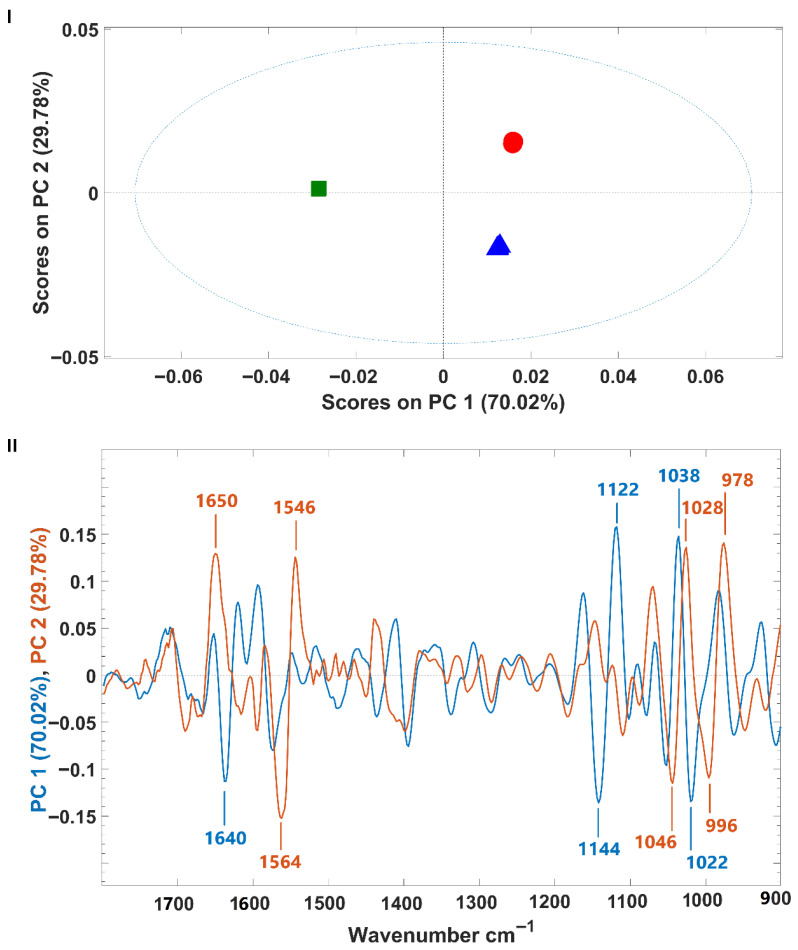
(**I**)—Scores plot from principal component analysis of infrared spectra of A and B *P. cruentum* samples (fermented or not). A samples are shown as blue triangles; B samples are red circles); fermented A samples are green squares; (**II**)—Loading plot of PC1 (blue, 70.02% variance) and PC2 (red, 29.78% variance), showing the wavenumber regions contributing most to sample separation.

*P. cruentum* infrared spectra were also subjected to a PCA. The model’s scores and the corresponding loading are presented in Figure 4. Similar to what was observed for *H. pluvialis*, samples were separated from each other along the first two principal components (PCs), which together encompass 99.80% of the spectral variance (PC1: 70.02%, PC2: 29.78%) (Figure 4I). However, for *P. cruentum* samples, the discrimination along PC1 was between fermented (negative part of PC1) and non-fermented samples (culture origins A and B) on the positive side of PC1. Samples A and B (from different growth origins) were discriminated from each other along PC2, which explains the lower spectral variability (almost 30%) (Figure 4I). These observations suggest that the microalgal culture origin has less impact on *P. cruentum* macromolecular composition than the fermentation process. Analysis of the model loadings showed that, for both PCs, the wavenumbers contributing most to sample discrimination fall within the protein region (1700–1500 cm^−1^) and the carbohydrate vibration ranges (1200–900 cm^−1^) (Figure 4II).

### 3.5. Microalgae Color Before and After Fermentation

For *H. pluvialis*, fermentation resulted in a significant increase in L* (lightness), indicating a lighter biomass (Table 5). There was also a shift in the hue angle from 27.8 ± 1.10° to 35.3 ± 2.20°, accompanied by a decrease in chroma from 5.00 ± 0.22 to 3.50 ± 0.15, suggesting a less saturated and slightly more yellow coloration. In contrast, fermentation of *P. cruentum* led to a decrease in L* values, indicative of darkening. Additionally, the hue angle decreased from 40.0 ± 1.10° to 34.3 ± 0.60°, and chroma declined from 10.13 ± 1.01 to 7.74 ± 0.81. This reduction in the hue angle likely reflects the microalgal color shifting from purple toward red–purple. These changes indicate that fermentation can alter the visual appearance of microalgal biomass, which may affect consumer acceptance when incorporated into food products.

### 3.6. Nutritional Composition of the Vegan Burger

The nutritional composition of vegan burgers with fermented and non-fermented biomasses of *H. pluvialis* and *P. cruentum* is presented in Table 6. A comparative analysis between the burger with non-fermented biomass of *H. pluvialis* (HB), and *P. cruentum* (PB), and the burger with fermented biomass (FHPB) highlights relevant nutritional trends. Protein content was the highest in HB and FHB (7.7 g/100 mg), lower in PB (6.5 ± 0.03 g/100 g), and was also high in the final product FHPB (7.3 ± 0.05 g/100 g). This suggests that the inclusion of 3% *H. pluvialis* and 3% *P. cruentum* did not dilute the burger‘s protein content, but rather contributed synergistically to the base mix. This aligns with literature, describing *H. pluvialis* as having a high protein content (29–45% dry weight (DW) [55]), while Martins et al. [56] found 42.5 ± 1.3 g/100 g DW protein in *P. cruentum*. Total fat content was higher in HB (7.6 ± 0.16 g/100 g) compared to PB (6.8 ± 0.2 g/100 g), with the final product FHPB stabilizing at 7.1 ± 0.04 g/100 g. The lipid content reflects the contribution of *H. pluvialis*, which is known for accumulating astaxanthin and other lipophilic compounds. Saturated fat remained constant across all formulations at around 1.0 g/100 g).

Carbohydrate levels were highest in PB (23.3 ± 0.1 g/100 g), lower in HB (22.4 ± 0.12 g/100 g), and marginally reduced in FHPB (22.0 ± 0.13 g/100 g), likely due to microbial consumption of fermentable sugars. Interestingly, fiber content increased in HPB (6.0 ± 0.03 g/100 g), suggesting a beneficial effect of fermentation and ingredient synergy, possibly enhancing the dietary fiber content by modifying complex polysaccharides [57]. A significant pH reduction from HB (5.56) to FHPB (4.34) confirms fermentation progress and improves product safety and stability. Ash content slightly decreased in FHPB (2.03 ± 0.01 g/100 g), while sodium remained stable around 0.56 ± 0.08 g/100 g, indicating balanced mineral retention during processing and consistent salt formulation.

### 3.7. Burger Color

The colorimetric parameters (L*, a*, b*, hue angle, and chroma) of the control vegan burger (C) and formulations incorporating *H. pluvialis*, *P. cruentum*, or both, in fermented and non-fermented forms, are presented in Table 7. The control sample exhibited the highest lightness (L* = 48.79 ± 3.90), indicating a comparatively lighter appearance. In contrast, samples with *H. pluvialis* alone (HB and FHB) showed significantly lower L* values (27.24 ± 1.79 and 27.78 ± 1.36, respectively), suggesting a darker visual profile. Formulations containing *P. cruentum* (PB and FPB) had intermediate lightness values (37.44 ± 2.64 and 39.16 ± 1.64), indicating that *P. cruentum* contributed to moderately darkened products. The a* values, representing the red–green color axis, were significantly influenced by microalgal biomass species. Samples with *P. cruentum* (PB and FPB) exhibited the highest redness values (19.23 ± 1.22 and 20.07 ± 1.24, respectively), which can be attributed to the presence of phycoerythrin, a red pigment characteristic of red microalgae [58]. In contrast, *H. pluvialis*-based samples showed lower a* values (HB: 7.04 ± 0.67; FHB: 7.49 ± 0.40), and the control sample had the lowest redness (2.81 ± 0.32), confirming the minimal red hue in the absence of microalgal pigments. Regarding the b* values (yellow–blue axis), the control sample showed the highest yellowness (11.89 ± 1.00), whereas formulations containing *H. pluvialis* alone had significantly lower b* values (around 4.15), indicating reduced yellow coloration. Samples containing *P. cruentum* (PB and FPB) exhibited intermediate b* values (7.0–7.6), suggesting that the pigment profile of *P. cruentum* included both red and yellow components. Hue angle (°), which reflects the overall perceived color, decreased significantly with the addition of microalgal biomass. The control had a hue angle of 76.73 ± 0.35°, corresponding to a yellowish hue. This value decreased markedly in PB and FPB (21.53 ± 0.48° and 19.25 ± 0.54°, respectively), consistent with a shift toward a redder appearance. Chroma values, indicative of color saturation or vividness, were highest in the PB and FPB samples (20.67 ± 1.30 and 21.26 ± 1.29, respectively), further confirming the strong pigment presence in *P. cruentum*. The control burger exhibited a moderate chroma (12.22 ± 1.02), while HB and FHB had the lowest saturation levels (8.2–8.6), indicating a duller color. Dual biomass formulations (HPB and FHPB) displayed intermediate chroma values (13.27), suggesting additive effects of both microalgal species.

**Figure 5:**
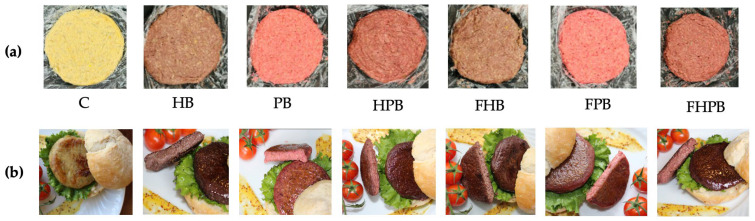
Photos of the vegan burgers with the different formulations; (**a**) crude, (**b**) cooked. C (control), HB: non-fermented *H. pluvialis*, PB: non-fermented *P. cruentum*, HPB: non-fermented *H. pluvialis P. cruentum*, FHB: *H. pluvialis* fermented, FPB: *P. cruentum* fermented, FHPB: fermented *H. pluvialis* and *P. cruentum*.

The color of the vegan burgers with the different formulations is shown in Figure 5.

### 3.8. Burger Texture

**Figure 6:**
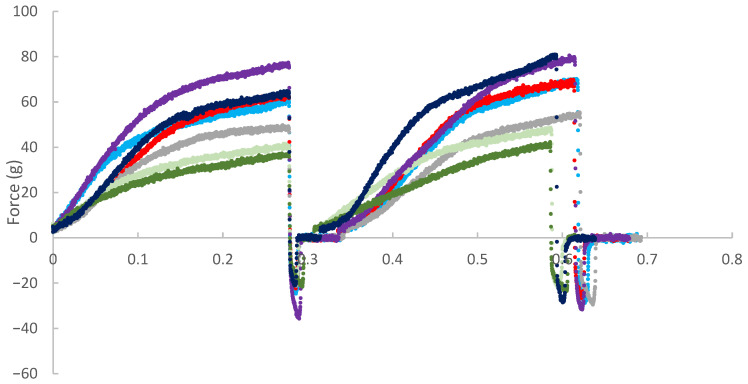
Texture profile of the different burger formulations enriched with microalgae.

The force (g) versus time (min) curves for the instrumental texture analyses of the different formulations are presented in Figure 6. The results show significant variation in the texture profiles among the seven formulations evaluated. The control formulation (C) exhibited intermediate hardness and good elasticity, as evidenced by shape recovery during the second compression cycle. The formulations containing non-fermented *P. cruentum* (P) and fermented *P. cruentum* (FP) showed the lowest maximum force values, indicating reduced mechanical resistance to compression. Additionally, these samples exhibited partial load loss early in the test (approximately 0.1 min), suggesting internal matrix fragility, early collapse, or structural rearrangement, which may be related to the presence of free water or a lower capacity for protein network formation. Formulations containing *H. pluvialis* (H, FH, HP, and FHP) exhibited higher rupture force, reflecting increased hardness and structural rigidity. In particular, samples FH and FHP showed the highest maximum force values (80–90 g). These formulations also demonstrated good structural recovery during the second compression cycle, indicating high cohesiveness and elasticity. The FHPB sample, containing both fermented *H. pluvialis* and *P. cruentum*, exhibited a texture profile characterized by high hardness, structural integrity under initial compression, and elevated resilience, traits considered desirable in alternative meat products. These findings suggest that combining microalgae may have a synergistic effect on product structure, promoting a more cohesive and resistant matrix.

Overall, the data indicate that the incorporation of *H. pluvialis*, either alone or in combination with *P. cruentum*, imparts superior textural characteristics compared to the other formulations, whereas the isolated use of *P. cruentum* may compromise the product’s structural integrity and chewability.

## 4. Discussion

### 4.1. Amino Acid *Profiles of the Fermented and Non-Fermented Biomasses*

The essential amino acid (EAA) ratio was similar across all samples, indicating that fermentation, although affecting absolute values, did not significantly compromise protein quality. This is particularly relevant for functional foods, as maintaining a high proportion of essential amino acids is crucial for dietary value. These findings align with previous studies, indicating that fermentation improves digestibility while preserving the essential amino acid balance in plant-based protein sources [50].

Valine levels were significantly higher in the non-fermented sample H (9.1 mg/100 mg), compared to all other groups (*p* < 0.01), including FH (6.6 mg/100 mg), FP (7.9 mg/100 mg), and P (6.6 mg/100 mg). This is consistent with literature reporting branched-chain amino acids (BCAAs) as dominant components in microalgae [59]. Glutamic acid, a non-essential amino acid known for its role in flavor and nitrogen metabolism, was particularly abundant in the H sample (4.9 mg/100 mg), in agreement with results for *H. pluvialis* described by Spolaore et al. [55].

Lysine, an essential amino acid often limited in plant-based diets, also showed statistically significant changes, with higher amounts in sample H (2.4 mg/100 mg), highlighting the nutritional potential of *H. pluvialis* as a protein source and supporting the notion that fermentation can modulate essential amino acid availability. Threonine and isoleucine were found at consistent levels across all treatments, further supporting the nutritional relevance of these algal species for human consumption. Some variation was observed; for example, a slight decrease in leucine in the fermented samples (FH and FP) may be attributed to microbial uptake of branched-chain amino acids during growth, as observed in fermentation studies. Enzymatic hydrolysis effects, where microbial and endogenous proteases alter free amino acid levels, could also contribute [60]. Alanine, often linked with stress metabolism and fermentation byproducts, was significantly elevated in FP (1.6 mg/100 mg) compared to P (1.3 mg/100 mg), potentially reflecting microbial deamination and transamination reactions.

The observed amino acid profiles are consistent with previous findings. For instance, *P. cruentum* is known for its high aspartic acid (11.2%), serine (8.1%), and glutamic acid (8.2%) content [61], while *H. pluvialis* is rich in glutamic acid and lysine [62]. The modest reduction in leucine and tyrosine in fermented samples may be explained by microbial decarboxylation or oxidative degradation during fermentation, a phenomenon previously described in lactic acid bacteria fermentations of plant matrices.

Taken together, these results demonstrate that microbial fermentation significantly alters the amino acid profile of *P. cruentum* and *H. pluvialis*, though not uniformly across species. Fermentation appears to have a protein-preserving or even enhancing effect in *P. cruentum*, while it leads to partial amino acid loss in *H. pluvialis*. These findings support the use of fermentation as a selective tool for modulating the nutritional and functional properties of algal biomass, aligning with trends in food biotechnology toward sustainable and enriched plant-based proteins.

### 4.2. Volatile Organic Compounds (VOCs)

In *P. cruentum*, a total of 42 VOCs were initially identified in the non-fermented biomass, including compounds such as *trans*-β-ionone, 4-ethyl-6-hepten-3-one, hexanal, and heptadienal. These are commonly associated with off-flavors, such as fishy or seaweed-like odors. Among the tested fermentations, the sample fermented with *Lb. plantarum* exhibited the most pronounced reduction in these undesirable VOCs, with complete elimination of 4-ethyl-6-hepten-3-one and a substantial decrease in *trans*-β-ionone (from 0.7263 mg/L to 0.1453 mg/L, Table 3). The decline of aldehydes such as hexanal and 2,4-heptadienal further supports the efficacy of fermentation in mitigating off-odors.

Similarly, in *H. pluvialis*, fermentation eliminated hexanal and significantly reduced heptadienal (from 0.4230 to 0.1747 mg/L, Table 2), both of which are key contributors to rancid and green notes originating from lipid oxidation.

Our findings on the reduction in fishy and green-smelling VOCs, such as hexanal, *trans*-β-ionone, and heptadienal, following *Lb. plantarum* fermentation, are consistent with previous studies on plant fermentations, where LAB activity mitigates undesirable odors through enzymatic transformations of aldehydes and terpenoids [63]. These modifications underline the sensorial improvements necessary for incorporating microalgae as food-grade ingredients.

### 4.3. Total Phenolic Content and Antioxidant Activity of H. pluvialis and P. cruentum

Fermentation significantly enhanced the TPC and antioxidant activity of both microalgae. For *H. pluvialis*, TPC increased more than sevenfold (from 0.1474 to 1.0795 mg GAE/100 mg DW), accompanied by improvements in ABTS (from 2.3967 to 3.2185 µmol TE/100 mg DW) and ORAC (from 43.3276 to 54.3239 µmol TE/100 mg DW) assays. Although DPPH activity was not determined due to color interference, the ABTS and ORAC assays confirmed enhanced antioxidant potential.

For *P. cruentum*, fermentation also improved antioxidant metrics, though at lower absolute levels. TPC rose from 0.0816 to 0.1802 mg GAE/100 mg DW, ABTS increased from 0.2644 to 0.3251 µmol TE/100 mg DW, and ORAC from 1.3806 to 3.1111 µmol TE/100 mg DW. Interestingly, DPPH values slightly declined post-fermentation, potentially due to changes in the phenolic profile or matrix interactions.

These enhancements are consistent with microbial bioconversion of phenolic precursors or the release of bound phenolics during fermentation. The increase in antioxidant capacity further supports the incorporation of fermented microalgae into functional food systems.

The marked rise in TPC and antioxidant capacity in *H. pluvialis* and *P. cruentum* aligns with previously reported effects of *Lb. plantarum* fermentation in various plant matrices, such as jujube juice and seaweed, where microbial release or synthesis of phenolic compounds elevated both TPC and radical-scavenging activity. Mechanistically, this effect is supported by LAB-mediated enzymolysis and acidification, processes known to liberate bound phenolics [64].

Moreover, the naturally high carotenoid and phenolic content of *H. pluvialis* correlates with antioxidant-rich oleoresins described by Ruiz-Dominguez et al. [65], where bioactivity results from synergistic effects between phenolics and astaxanthin. Our results show that fermentation further enhances this profile, offering potential for functional food applications.

Seaweed species like *Gelidium* sp. and *Eucheuma cottonii*, which experienced enhanced TPC and radical scavenging post-fermentation, corroborate the effect of LAB [66]. In plant systems like broccoli puree, *Lb. plantarum* has demonstrated to elevate phenolic levels by releasing bound compounds [67]. These improvements are likely due to microbial enzymatic activity that liberates phenolics and possibly synthesizes novel antioxidant metabolites.

### 4.4. FTIR and PCA-Monitored Macromolecular Changes in H. pluvialis and P. cruentum

FTIR analyses of *H. pluvialis* and *P. cruentum* highlighted biochemical modifications associated with growth phases and fermentation. In *H. pluvialis*, the aplanospore stage exhibited higher lipid and carotenoid content than the palmella stage, as evidenced by increased absorption at 2925, 2850, and 3010 cm^−1^. The 1740 cm^−1^ band, characteristic of astaxanthin, was more pronounced in aplanospore samples, corroborating carotenoid accumulation during maturation.

Spectral PCA confirmed distinct clustering of palmella, aplanospore, and fermented aplanospore samples. The carbohydrate absorption region (1200–900 cm^−1^) was decisive in PC1-based separation (palmella vs. aplanospore), consistent with known shifts in carbohydrate composition from cellulose to mannose during differentiation. PC2 loadings, particularly at 1626 and 1046 cm^−1^, differentiated fermented samples, suggesting rearrangements in protein and sugar structures due to microbial activity.

In contrast, FTIR spectra of *P. cruentum* were less affected by culture growth conditions but showed significant changes upon fermentation. PCA showed that fermentation drove separation along PC1, with protein and carbohydrate regions (1700–1500 cm^−1^ and 1200–900 cm^−1^) contributing most. Compared to *H. pluvialis*, *P. cruentum* showed lower lipid and sugar intensities and higher protein signals, reflecting interspecies differences in biochemical composition.

These results confirm that fermentation exerts a greater influence on the macromolecular fingerprint of *P. cruentum* than cultivation conditions.

FTIR spectra reveal that fermentation leads to clear biochemical changes in the microalgal matrix, particularly in carbohydrate and protein domains. These structural shifts are consistent with findings from fermented plant-based foods, where microbial action alters cell wall polysaccharides and protein structures [68]. For *H. pluvialis*, the increased lipid and carotenoid signatures observed in the aplanospore stage may indicate microbial stability or enhanced compound accessibility. Although this warrants further investigation, similar enhancements in carotenoid bioavailability have been reported post-fermentation in other vegetable-based substrates.

Overall, FTIR-PCA analyses demonstrated clear discrimination between growth phases and fermentation states, with carbohydrate- and protein-related bands playing central roles. These observations align with documented structural changes in polysaccharide composition during fermentation of both plant and algal materials. [67]. The persistence of elevated lipid and carotenoid absorption in *H. pluvialis* aplanospore post-fermentation suggests that fermentation may help preserve, or even enhance, bioactive compound profiles, supporting their potential in functional food development.

### 4.5. Color of H. pluvialis and P. cruentum Before and After Fermentation

Fermentation induced significant alterations in the color parameters of both *H. pluvialis* and *P. cruentum*, with potential implications for product formulation and consumer acceptance. In *H. pluvialis*, L* values increased from 9.51 ± 0.51 to 12.32 ± 0.38, indicating a lighter appearance. Concurrently, chroma values decreased from 5.00 ± 0.22 to 3.50 ± 0.15, suggesting a less saturated color, while the hue angle shifted from 27.8 ± 1.10° to 35.3 ± 2.20°, reflecting a transition to a more yellowish tone.

In contrast, *P. cruentum* showed decreased L* values from 19.11 ± 1.29 to 16.77 ± 1.34, indicating darkening, and a reduction in chroma (from 10.13 ± 1.01 to 7.74 ± 0.81). The hue angle shifted from 40.0 ± 1.10° to 34.3 ± 0.60°, suggesting changes in pigment composition or degradation of phycobiliproteins as a result of fermentation.

These color changes are particularly relevant in the context of food product development, where visual attributes strongly influence consumer perception and acceptance.

The observed color shifts—lightening in *H. pluvialis* and darkening in *P. cruentum*—likely result from pigment transformation or degradation, a phenomenon similarly reported in LAB fermentation of seaweed, where pigment changes in ΔL* and ΔC* values and correlated with alterations in pigment content and functional properties [69,70]. The enhanced red and brown hues observed in both fermented *P. cruentum* and *H. pluvialis*, respectively, are generally favorable, as they mimic the appearance of cooked meat products. From a sensory standpoint, darker tones achieved through fermentation may enhance the authenticity and appeal of alternative protein products. Such shifts are considered favorable in consumer-facing applications, particularly in alternative meat analogs, where color plays a crucial role in perceived quality and palatability [71,72].

Overall, these fermentation-induced color modifications highlight the importance of monitoring visual parameters during the development of microalgae-based food ingredients, ensuring they align with consumer expectations and sensory standards in functional food products.

### 4.6. Burger Nutritional Composition

Statistical analysis of the nutritional components across the seven vegan patty formulations revealed notable differences in nutritional components. Saturated fatty acids remained consistent among all groups (*p* > 0.05), indicating that fermentation and formulation had no significant impact on the overall saturated fat profile. In contrast, total carbohydrates varied significantly (*p* < 0.05), with the control (C) group showing higher carbohydrate content than FPB and FHPB. Dietary fiber content also showed significant differences, with HPB, FPB, and FHB exhibiting higher levels, while FHPB had substantially lower content (*p* < 0.05). Protein content differed markedly across groups (*p* < 0.01), with HB, FHB, HPB, and FPB showing higher levels compared to C and PB, highlighting the protein-enhancing potential of both formulation and fermentation. Sodium levels were not significantly different among groups (*p* > 0.05); however, sodium chloride content was significantly reduced in FHB compared to FPB (*p* < 0.05), potentially reflecting fermentation-associated sodium utilization or redistribution. Moisture content also showed strong statistical differences (*p* < 0.01), with higher values observed in FPB and FHPB than in C and HB. For ash content, PB had significantly higher mineral levels than FHPB and HPB (*p* < 0.05). Lastly, pH analysis revealed substantial differences (*p* < 0.001), with the control (C) and HB exhibiting the highest pH, while fermented formulations (e.g., FPB, FHPB) had significantly lower, more acidic values. These changes underline the influence of fermentation and added biofunctional ingredients on the overall composition of the products. These findings reflect the compositional shifts induced by formulation changes and processing methods across the product variants.

Table 6 presents the detailed nutritional composition of vegan burgers enriched with fermented and non-fermented biomasses of *H. pluvialis* and *P. cruentum*. Fermentation consistently increased protein levels in both species, particularly in *H. pluvialis*, likely due to microbial biomass contribution or enhanced protein extractability. Conversely, a slight reduction in carbohydrate and sugar contents was noted in fermented samples, which may be attributed to microbial utilization of these components as energy sources during fermentation. Fiber content, especially soluble fiber, tended to increase post-fermentation, likely as a result of partial polysaccharide hydrolysis, making bound fiber more bioaccessible or analytically detectable. These compositional changes also influenced the energy content of the burger, with fermented variants typically showing lower caloric values compared to their non-fermented counterparts. However, caution is warranted in interpreting these data due to methodological differences in nutrient quantification. For example, protein quantification via the Kjeldahl method may yield inflated values relative to chromatographic methods such as HPLC, due to possible nitrogen loss or alteration during analysis.

The development of microalgae-enriched vegan burgers aligns with growing consumer interest in sustainable and health-oriented alternatives to conventional meat. The base ingredients, chickpeas, lentils, and quinoa, offer a nutritionally synergistic matrix. Legumes contribute plant protein, complex carbohydrates, and dietary fiber, with a high lysine content, while quinoa complements this by providing all essential amino acids, including methionine and cysteine, often limited in legumes [73]. Furthermore, quinoa adds minerals such as magnesium, iron, and zinc, along with polyphenols and flavonoids known for their antioxidant properties [74]. This combination supports a complete protein profile and may help maintain lean mass in plant-based diets. The dietary fiber content from these ingredients may contribute to improved gut health, satiety, and glycemic control, supporting recommendations from the EFSA and WHO for fiber intake (25–30 g/day) [75].

The addition of *H. pluvialis* and/or *P. cruentum* further enhances the functional value of the patties. *H. pluvialis* is a rich natural source of astaxanthin, a potent carotenoid with anti-inflammatory, antioxidant, and neuroprotective properties [76]. Clinical and preclinical studies have demonstrated its efficacy in improving oxidative stress markers and lipid profiles [77]. *P. cruentum* contributes sulfated polysaccharides with immunomodulatory, antiviral, and antioxidant activities [78], along with phycoerythrin and fatty acids such as eicosapentaenoic acid (EPA), which support cardiovascular and anti-inflammatory health [79]. Fermentation of microalgal biomass may further enhance the nutrient bioavailability and digestibility by reducing antinutritional factors and increasing B-vitamin levels and bioactive peptide content [57].

From a sustainability perspective, microalgae cultivation is highly efficient, requiring minimal land and freshwater, while offering carbon sequestration potential. Integrating microalgae into plant-based food systems aligns with the EAT-Lancet Commission’s vision for a planetary health diet [80]. Replacing animal-derived proteins with plant- and algae-based alternatives can substantially reduce greenhouse gas emissions, conserve biodiversity, and promote more resilient food systems [81].

### 4.7. Burger Color

Fermentation and the incorporation of microalgal biomass caused notable changes in the color profile of the vegan burger formulations. The control burger (C) had the highest lightness and hue angle near 77°, corresponding to a golden-beige appearance. In contrast, burgers containing fermented microalgae, particularly *P. cruentum*, appeared darker, redder, and more chromatically saturated. These color modifications diverged from those observed in the isolated microalgal biomasses, highlighting the influence of matrix interactions, heat application, and food processing on pigment behavior and expression. From a sensory perspective, these color changes may influence consumer perception in both positive and negative ways. Redder hues may enhance the perceived similarity to traditional meat products, whereas darker tones could be misinterpreted as signs of overcooking or artificial additives. Thus, optimizing the visual attributes is crucial in product development.

Among the tested formulations, *P. cruentum* incorporation, especially in its fermented form, significantly enhanced the redness (a*) and chroma values of the burgers. This effect aligns with previous findings that red microalgae, such as *P. cruentum*, are rich in water-soluble phycobiliproteins, primarily phycoerythrin, which contribute vivid coloration [53]. In contrast, *H. pluvialis*, known for its astaxanthin content, tended to yield darker yet less saturated colors [82]. Fermentation generally intensified pigmentation across both species, likely due to pigment stabilization or increased pigment extractability, possibly facilitated by microbial degradation of cell wall structures.

### 4.8. Burger Texture

The texture profile results of the seven burger formulations enriched with *H. pluvialis* and *P. cruentum* revealed notable differences across samples, driven by ingredient composition and matrix interactions. Liu et al. [50] have shown that the use of microalgae, particularly *H. pluvialis*, can influence key textural properties, namely hardness and elasticity, in extruded plant-based products. In alignment with these findings, the current study demonstrated that formulations containing *H. pluvialis* (H, FH, HP, and FHP) exhibited higher initial compression force and superior structural recovery, indicative of enhanced cohesiveness and elasticity. Formulations with isolated or combined *H. pluvialis* (FH and FHP) were especially characterized by improved structural resilience. This is consistent with the hypothesis that the robust cell walls of this microalga contribute to fibrous structure formation and increased elasticity in meat analogues, as previously described by Liu et al. [50]. In contrast, formulations containing only *P. cruentum* (P and FP) exhibited lower hardness and an early onset of structural failure (evident from partial load drop at approximately 0.1 min), indicating reduced mechanical integrity. This observation may be associated with the presence of unstructured liquid components in *P. cruentum*, a phenomenon previously linked to diminished textural stability in high-microalgae formulations [83]. Additionally, Benković et al. [84] highlighted the role of microalgal proteins in conferring emulsifying and gelling properties, which can enhance mouthfeel and chewability, factors also relevant in the current burger formulations. While the incorporation of microalgae appears to offer specific textural benefits, particularly through the modulation of elasticity and cohesiveness, it should be noted that high concentrations of algal biomass may negatively impact color and flavor. These potential drawbacks, often reported in the literature, underscore the importance of balancing functional benefits with sensory attributes. In this study, sensory perception was not quantitatively assessed, representing a critical area for future research. Further work should explore how textural improvements from microalgae incorporation translate to consumer acceptability in real-world applications.

### 4.9. Implementation Considerations

The incorporation of *Haematococcus pluvialis* and *Porphyridium cruentum* into plant-based burger formulations brings multiple functional benefits, including natural coloration, aroma modulation, textural influence, and acidity balance. These attributes are particularly relevant in the context of clean-label product development, where there is growing consumer demand for natural alternatives to synthetic colorants, flavorings, and texturizers. Specifically, the reddish pigments (astaxanthin in *H. pluvialis* and phycoerythrin in *P. cruentum*) contribute a visually appealing hue, while fermentation and cell wall components introduce mild acidity and umami-like notes, which can help mask or modify off-flavors typically associated with plant proteins or microalgal biomass.

However, the cost and scalability of producing these microalgal ingredients currently pose a challenge for large-scale commercialization. Microalgal cultivation, harvesting, and processing remain more expensive than traditional additives such as synthetic colorants, flavor enhancers, or acidity regulators. Despite this, the multifunctional nature of these microalgae, delivering color, aroma, texture, and nutritional enrichment in a single ingredient, offers a compelling case for their integration into premium clean-label or functional food segments, where added value can justify higher ingredient costs. This premium plant-based burger could cost 30% to 40% more than a commercial vegan burger.

Moreover, the microalgal biomass contributes not only sensory attributes but also bioactive compounds (e.g., antioxidants, polysaccharides, essential fatty acids), which may provide health-related marketing opportunities not possible with conventional additives. As microalgae cultivation technologies become more efficient and sustainable (e.g., through photobioreactors, co-product valorization, or use of waste streams), cost barriers are expected to decline, improving the economic feasibility of these ingredients for wider food industry applications. Raw pulses used as ingredients of this plant-based burger are low-cost.

In conclusion, while microalgae-derived ingredients may not yet compete with conventional additives purely on cost, their natural origin, multifunctionality, and nutritional value position them as promising candidates for use in next-generation plant-based products. Their suitability will be especially relevant in clean-label formulations targeting health-conscious and environmentally aware consumers, making them a strategic option for differentiated market segments rather than direct substitutes for low-cost synthetic additives.

## 5. Conclusions

This study demonstrated that *Lactiplantibacillus plantarum* fermentation effectively modulates the biochemical, functional, and sensory characteristics of *Porphyridium cruentum* and *Haematococcus pluvialis*, enhancing their potential as high-value ingredients for food applications. Fermentation significantly altered amino acid profiles, volatile organic compounds (VOCs), antioxidant activity, total phenolic content, and chromatic attributes, with effects varying by species. Notably, fermentation reduced off-flavor volatile organic compounds (VOCs), enhanced antioxidant capacity and phenolic content, and induced favorable shifts in color parameters, particularly in red hue intensity and saturation.

FTIR spectroscopy and multivariate analysis (PCA) confirmed profound macromolecular transformations in both species, especially within carbohydrate and protein domains. These modifications were more strongly influenced by fermentation than by growth cultivation conditions. These findings highlight the potential of *Lb. plantarum* fermentation in shaping the biochemical fingerprint of microalgal biomass. Observed changes parallel benefits documented in other fermented food systems and underscore the value of fermented microalgae as a functional food ingredient.

Incorporation of fermented and non-fermented *H. pluvialis* and *P. cruentum* into a vegan burger matrix further illustrated their functional value. Both microalgae contributed key nutritional components, including plant-derived protein, dietary fiber, essential amino acids, and bioactive compounds such as astaxanthin, EPA, and sulfated polysaccharides. Fermentation enhanced pigment extractability and stability, leading to intensified redness and saturation, particularly from *P. cruentum*, while *H. pluvialis* supported firmer texture and structural integrity in the burgers.

This study prioritized establishing cultivation protocols and conducting detailed physicochemical and compositional analyses to support the proof-of-concept stage. The potential prototype remains experimental, particularly regarding *Porphyridium cruentum*, due to regulatory restrictions, which render conventional sensory testing inappropriate at this stage. Instead, the researchers employed instrumental techniques, including GC-MS analysis of volatile compounds, objective color measurement, and texture profiling, to generate reliable, ethically sound data on sensory-related attributes. While these methods cannot replace full consumer sensory evaluation, they provide a robust foundation for assessing the burger’s functional potential, with comprehensive sensory studies planned once safety (microbiological and toxicological analysis) and regulatory requirements are met. Namely, if fermentation is not well controlled, some harmful components such as nitrite and biogenic amines may be produced. This phenomenon is not exclusive to microalgae-based products; similar accumulations of nitrite and biogenic amines have been documented in plant-based fermented vegetables [85], leading to potential safety concerns. Nevertheless, Ribeiro et al. [86] reported no biogenic amines produced in fermentation with the same strain of *Lb. Plantarum* used in this study.

Overall, this work highlights the potential of *Lb. plantarum*-mediated fermentation to improve the nutritional quality, sensory appeal, and functional versatility of microalgal biomass. The observed improvements parallel benefits reported in other fermented food systems and underscore the relevance of microalgae as adaptable, functional food ingredients. This study aligns with global dietary and sustainability goals by promoting health-oriented and eco-friendly innovations. Future research should focus on sensory panel assessments and bioavailability studies to validate consumer acceptance and ensure commercial feasibility. In addition, commercialization will require further safety validation and regulatory pathways.

## Figures and Tables

**Table 1:** Amino acid profiles (mg/100 mg protein) of *H. pluvialis* and *P. cruentum* biomasses (fermented and non-fermented).

Amino Acid	Fermented *H. pluvialis* (FH)	Fermented *P. cruentum* (FP)	*H. pluvialis* (H)	*P. cruentum* (P)
Aspartic acid	1.742 ± 0.005 ^a^	1.959 ± 0.021 ^ab^	2.349 ± 0.006 ^c^	2.013 ± 0.030 ^b^
Glutamic acid	3.608 ± 0.026 ^a^	3.938 ± 0.011 ^ab^	4.898 ± 0.128 ^c^	3.721 ± 0.060 ^b^
Serine	0.916 ± 0.143 ^a^	0.812 ± 0.005 ^ab^	1.059 ± 0.010 ^c^	0.483 ± 0.683 ^b^
Glycine	1.593 ± 0.237 ^a^	1.384 ± 0.015 ^ab^	1.696 ± 0.009 ^c^	1.376 ± 0.010 ^b^
Threonine	1.252 ± 0.040 ^a^	1.274 ± 0.011 ^ab^	1.605 ± 0.001 ^c^	1.273 ± 0.007 ^b^
Arginine	1.047 ± 0.081 ^a^	1.001 ± 0.002 ^a^	1.536 ± 0.020 ^a^	1.374 ± 0.117 ^a^
Alanine	1.312 ± 0.093 ^a^	1.644 ± 0.177 ^a^	1.776 ± 0.230 ^a^	1.325 ± 0.156 ^a^
Tyrosine	0.560 ± 0.044 ^a^	0.689 ± 0.022 ^a^	0.773 ± 0.004 ^a^	0.649 ± 0.003 ^a^
Valine	6.554 ± 0.513 ^a^	7.947 ± 0.025 ^ab^	9.106 ± 0.001 ^c^	6.555 ± 0.352 ^b^
Phenylalanine	1.293 ± 0.101 ^a^	1.491 ± 0.009 ^ab^	1.822 ± 0.001 ^c^	1.465 ± 0.001 ^b^
Isoleucine	3.351 ± 0.262 ^a^	3.958 ± 0.026 ^ab^	4.837 ± 0.013 ^c^	4.226 ± 0.109 ^b^
Leucine	0.623 ± 0.048 ^a^	0.509 ± 0.010 ^a^	0.989 ± 0.005 ^a^	0.992 ± 0.083 ^a^
Lysine	1.879 ± 0.147 ^a^	2.049 ± 0.022 ^a^	2.383 ± 0.007 ^a^	1.736 ± 0.013 ^a^
Total AA	25.730 ^c^	28.654 ^b^	34.832 ^a^	27.188 ^b^
EAA Ratio (%)	58.11 ^a^	60.13 ^a^	59.55 ^a^	59.76 ^a^

Different letters in each row indicate significant differences (*p* < 0.05).

**Table 2:** Concentrations (mg/mL) of the main volatile organic compounds of *H. pluvialis* before and after fermentation.

Sample	Compound Concentration (mg/L)						
	Hexanal	1-Pentanol	Octanone	1-Hexanol	E-2,4-Heptadienal	8-Heptadecene	1-Penten-3-ol
H	0.3113 ± 0.0740 ^b^	0 ^a^	0.17512 ± 0.0113 ^b^	0 ^a^	0.4230 ± 0.0312 ^b^	0.2274 ± 0.0176 ^a^	0.1487 ± 0.0117 ^b^
FH	0 ^a^	0.1220 ± 0.0024 ^b^	0 ^a^	0.1352 ± 0.0024 ^b^	0.1747 ± 0.0323 ^a^	6.3720 ± 0.3720 ^b^	0.1408 ± 0.0022 ^ab^

Different letters in each column mean significant differences (*p* < 0.05). Three replicates.

**Table 3:** Concentrations (mg/mL) of the main volatile organic compounds of *P. cruentum* before and after fermentation.

Sample	Compound Concentration (mg/L)				
	2-Hexene 3,5, 5-timethyl	E-2-Octenal	*trans*-β-lonone	Hexanal	2,4-Heptadienal
P	1.6982 ± 0.1056 ^b^	1.6982 ± 0.0065 ^b^	0.7263 ± 0.0531 ^b^	0.1654 ± 0.0042 ^b^	0.0521 ± 0.0073 ^b^
FP	0 ^a^	0 ^a^	0.1453 ± 0.0026 ^a^	0.0313 ± 0.0007 ^a^	0 ^a^

Different letters in each column mean significant differences (*p* < 0.05). Three replicates.

**Table 4:** Total phenolic content and antioxidant activity of *H. pluvialis* and *P. cruentum* before and after fermentation with *L. plantarum*.

Sample	TPC (mg GAE/100 mg DW)	ABTS	DPPH (μmol TE/100 mg DW)	ORAC
Fermented *H. pluvialis*	1.0795 ± 0.2299 ^a^	3.2185 ± 0.3526 ^a^	-	54.3239 ± 1.7887 ^a^
Non-fermented *H. pluvialis*	0.1474 ± 0.0056 ^b^	2.3967 ± 0.1299 ^b^	-	43.3276 ± 2.9053 ^b^
Fermented *P. cruentum*	0.1802 ± 0.0257 ^c^	0.2644 ± 0.0005 ^c^	0.0836 ± 0.0037 ^a^	3.1111 ± 0.1273 ^c^
Non-fermented *P. cruentum*	0.0816 ± 0.0032 ^d^	0.3251 ± 0.0285 ^d^	0.1368 ± 0.0026 ^b^	1.3806 ± 0.1215 ^d^

Different letters in each column mean significant differences (*p* < 0.05). Three replicates.

**Table 5:** Color parameters of *H*. *pluvialis* and *P. cruentum* before and after fermentation with *L. plantarum*.

Sample	L*	a*	b*	Hue (°)	Croma
Non-fermented *H. pluvialis*	9.51 ± 0.51 ^a^	2.33 ± 0.10 ^b^	4.48 ± 0.12 ^b^	27.8 ± 1.10 ^a^	5.00 ± 0.22 ^b^
Fermented *H. pluvialis*	12.32 ± 0.38 ^b^	2.00 ± 0.08 ^a^	2.50 ± 0.10 ^a^	35.3 ± 2.20 ^bc^	3.50 ± 0.15 ^a^
Non-fermented *P. cruentum*	19.11 ± 1.29 ^d^	6.00 ± 0.50 ^d^	8.20 ± 0.70 ^d^	40.0 ± 1.10 ^d^	10.13 ± 1.01 ^d^
Fermented *P. cruentum*	16.77 ± 1.34 ^c^	5.50 ± 0.45 ^c^	6.00 ± 0.55 ^c^	34.3 ± 0.60 ^b^	7.74 ± 0.81 ^c^

Different letters in each column mean significant differences (*p* < 0.05). Ten replicates.

**Table 6:** Nutritional composition of the vegan burgers, control, and with incorporated *H. pluvialis* and/or *P. cruentum*, biomasses fermented/non-fermented.

Component (g/100 g)	C	HB	PB	FHB	FPB	HPB	FHPB
Total Fat	7.1 ± 0.11 ^b^	7.6 ± 0.16 ^a^	6.8 ± 0.20 ^bc^	7.1 ± 0.06 ^b^	6.4 ± 0.08 ^c^	7.1 ± 0.12 ^b^	7.1 ± 0.04 ^b^
Saturated Fatty Acids	1.1 ± 0.15 ^ab^	1.1 ± 0.11 ^ab^	1.0 ± 0.16 ^b^	1.0 ± 0.16 ^b^	1.0 ± 0.09 ^b^	1.1 ± 0.09 ^b^	1.3 ± 0.03 ^ab^
Total Carbohydrates	24.0 ± 0.12 ^a^	22.4 ± 0.12 ^b^	23.3 ± 0.10 ^ab^	22.9 ± 0.10 ^ab^	21.9 ± 0.14 ^c^	22.3 ± 0.20 ^b^	22.0 ± 0.13 ^c^
Total Dietary Fiber	5.6 ± 0.11 ^b^	5.5 ± 0.19 ^b^	5.5 ± 0.16 ^b^	5.8 ± 0.12 ^ab^	5.9 ± 0.02 ^ab^	6.0 ± 0.03 ^a^	5.2 ± 0.04 ^c^
Protein	6.6 ± 0.09 ^c^	7.7 ± 0.02 ^a^	6.5 ± 0.03 ^c^	7.7 ± 0.01 ^a^	7.5 ± 0.14 ^ab^	7.6 ± 0.05 ^ab^	7.3 ± 0.05 ^b^
Sodium	0.58 ± 0.13 ^a^	0.55 ± 0.03 ^ab^	0.58 ± 0.13 ^a^	0.50 ± 0.13 ^b^	0.59 ± 0.14 ^a^	0.56 ± 0.04 ^ab^	0.56 ± 0.08 ^ab^
Sodium Chloride	1.45 ± 0.09 ^a^	1.38 ± 0.01 ^bc^	1.45 ± 0.04 ^a^	1.25 ± 0.13 ^c^	1.48 ± 0.05 ^a^	1.4 ± 0.13 ^b^	1.4 ± 0.17 ^b^
Moisture	60.2 ± 0.18 ^b^	60.1 ± 0.17 ^b^	61.2 ± 0.19 ^ab^	60.2 ± 0.13 ^b^	62.1 ± 0.03 ^a^	60.9 ± 0.06 ^ab^	61.6 ± 0.03 ^a^
Ash	2.13 ± 0.19 ^ab^	2.19 ± 0.16 ^ab^	2.22 ± 0.11 ^a^	2.15 ± 0.19 ^ab^	2.17 ± 0.07 ^ab^	2.09 ± 0.10 ^b^	2.03 ± 0.17 ^b^
pH	5.93 ± 0.08 ^a^	5.56 ± 0.18 ^ab^	4.77 ± 0.09 ^b^	4.36 ± 0.14 ^c^	4.06 ± 0.08 ^c^	4.75 ± 0.06 ^b^	4.34 ± 0.03 ^c^
Energetic value (kcal)	183.4	182.1	177.7	180.9	174.1	180.8	180.8

Different letters in each row mean significant differences (*p* < 0.05). Three replicates.

**Table 7:** Color parameters of the vegan burgers, control, and with incorporated *H. pluvialis* and/or *P. cruentum*, biomasses fermented/non-fermented.

Burger	L*	a*	b*	Hue (°)	Chroma
C	48.79 ± 3.90 ^a^	2.81 ± 0.32 ^d^	11.89 ± 1.00 ^a^	76.73 ± 0.35 ^a^	12.22 ± 1.02 ^b^
HB	27.24 ± 1.79 ^d^	7.04 ± 0.67 ^c^	4.15 ± 0.33 ^d^	30.56 ± 2.46 ^b^	8.18 ± 0.66 ^c^
PB	37.44 ± 2.64 ^b^	19.23 ± 1.22 ^a^	7.58 ± 0.47 ^b^	21.53 ± 0.48 ^d^	20.67 ± 1.30 ^a^
HPB	30.64 ± 1.37 ^c^	12.12 ± 0.51 ^b^	5.41 ± 0.33 ^c^	24.02 ± 0.60 ^c^	13.27 ± 0.59 ^b^
FHB	27.78 ± 1.36 ^c,d^	7.49 ± 0.40 ^c^	4.17 ± 0.26 ^d^	29.14 ± 0.96 ^b^	8.57 ± 0.45 ^c^
FPB	39.16 ± 1.64 ^b^	20.07 ± 1.24 ^a^	7.01 ± 0.40 ^b^	19.25 ± 0.54 ^e^	21.26 ± 1.29 ^a^
FHPB	30.65 ± 1.38 ^c^	12.20 ± 0.60 ^b^	5.20 ± 0.23 ^c^	23.09 ± 0.31 ^c^	13.27 ± 0.63 ^b^

Different letters in each column mean significant differences (*p* < 0.05). Ten replicates.

## Data Availability

The original contributions presented in the study are included in the article, further inquiries can be directed to the corresponding authors.
